# In Vitro Antibacterial and Antioxidative Activity and Polyphenolic Profile of the Extracts of Chokeberry, Blackcurrant, and Rowan Berries and Their Pomaces

**DOI:** 10.3390/foods13030421

**Published:** 2024-01-28

**Authors:** Kadrin Meremäe, Piret Raudsepp, Linda Rusalepp, Dea Anton, Uko Bleive, Mati Roasto

**Affiliations:** 1 Chair of Veterinary Biomedicine and Food Hygiene, Institute of Veterinary Medicine and Animal Sciences, Estonian University of Life Sciences, Fr. R. Kreutzwaldi 56/3, 51006 Tartu, Estonia; piret.raudsepp@emu.ee (P.R.); dea.anton@emu.ee (D.A.); mati.roasto@emu.ee (M.R.); 2 Polli Horticultural Research Centre, Chair of Horticulture, Institute of Agricultural and Environmental Sciences, Estonian University of Life Sciences, Uus 2, 69108 Polli, Estonia; uko.bleive@emu.ee

**Keywords:** antibacterial effect, MIC, antioxidative activity, phenolic compounds, chokeberry, blackcurrant, rowan berries, berry pomaces

## Abstract

The chemical composition of berries and berry pomaces is diverse, containing polyphenolic components that may have both antibacterial and antioxidative properties. In the present study, in vitro antibacterial effect of the extracts of chokeberry, blackcurrant, and rowan berries and berry pomaces against *L. monocytogenes*, *S. aureus*, *E. coli*, and *C. jejuni* was studied. In addition, the polyphenolic profile and antioxidant activity of these extracts were investigated. The polyphenolic profiles in the aqueous and 30% ethanolic extracts were determined chromatographically by HPLC-MS, and the total polyphenol content was estimated spectrophotometrically by HPLC-DAD-UV. The minimal inhibition concentrations (MICs) of the extracts against tested bacteria were determined by the broth microdilution method. The content of total polyphenols was highest and good antioxidative properties of the extracts were determined for chokeberry and blackcurrant berries and their pomaces. The highest proportions of total quercetin derivatives and anthocyanins were found in the extracts of chokeberry berry/pomace and blackcurrant berry/pomace, respectively. The sensitivity of tested microbes to the extracts of berries and berry pomaces was as follows: *S. aureus* > *L. monocytogenes* > *E. coli* and *C. jejuni.* In vitro antibacterial activity of tested extracts depended on the extraction solvent, mainly for the ethanolic extracts. Findings suggest that chokeberry and blackcurrant berries and their pomaces can be used as a good source of polyphenols with antioxidative properties, and they also have antibacterial activity against some foodborne pathogenic bacteria. It is important that the valuable compounds are extracted from juice press residues before their disposal.

## 1. Introduction

In recent years, there has been an increasing trend in food technology to increase the use of various plant-origin materials, including vegetable, fruit, and berry pomaces, in the composition of food of animal origin [1,2,3]. The reasons for this are food safety and durability-improving properties [1] and the potential health benefits of plant-derived bioactive components to human health [4]. Also, it is in good agreement with the principles of the circular bioeconomy, because the use of berry pomaces reduces food waste and provides added value to the final products [5]. 

Among the berries, chokeberry [6], blackcurrant [7], and rowan berries [8] are rich sources of polyphenolic compounds, primarily anthocyanins, which act as good antioxidants for the human body [9]. In addition, polyphenolic compounds also have antimicrobial [10,11] and anti-inflammatory properties [12]. Bioactive compounds in berries include phenolic compounds such as phenolic acids, flavonoids, e.g., anthocyanins and flavonols, and tannins [13]. Berry pomaces, a by-product of juice production, are also of interest because they contain valuable phenolic compounds [14] and therefore have a great potential as a good dietary supplement in foods [15]. According to Heinonen [16] and Klavins et al. [14], the content of total polyphenols in berries is uneven, e.g., 10% in berry pulp, 28–35% in berry skin, and 60–70% in berry seeds, therefore the berry pomace, which contains berry seeds and skins, is a valuable source of polyphenols. According to our knowledge, the amount of berry pomaces is dependent on variety but is approximately 15–20% of the weight of fresh berries.

Several studies [17,18,19] have shown the antibacterial properties of berry pomaces against Gram-negative and Gram-positive bacteria. Furthermore, the good antibacterial properties of polyphenolic compounds and their use in food composition can contribute to the microbiological safety and quality of ready-to-eat foods of animal origin by inhibiting of spoilage microorganisms and essential foodborne pathogens [20]. Therefore, the possible antimicrobial properties of both berries and berry pomaces would be a compelling argument for the food industry for their wider use in food production. However, from the point of view of the development of health-promoting foods, it is also of interest for food producers to use plant materials incl. berries and their pomaces that have proven antibacterial properties in both in vitro and in vivo studies.

The main aim of this work is to determine in vitro antibacterial effect of aqueous and 30% ethanolic extracts of chokeberry, blackcurrant, and rowan berries and their pomaces against the growth of *L. monocytogenes*, *S. aureus*, *E. coli*, and *C. jejuni*. In addition, the polyphenolic profile and antioxidant activity of extracts of selected berries and their pomaces were investigated.

## 2. Materials and Methods

### 2.1. Plant Material and Preparation of Extracts

Chokeberry (*Aronia melanocarpa* (Michx.) Elliott), blackcurrant (*Ribes nigrum* L.), and rowan (*Sorbus aucuparia* L.) berries were harvested in Tartu County, Estonia. The berry pomaces were obtained as a by-product of berry juice production using the SmegSJF01CREU low-speed juicer (Smeg S.p.A, Guastalla, Italy) at the Polli Horticultural Research Centre, Estonian University of Life Sciences (Polli, Estonia). Plant materials were dried at 50 °C in a condensation dryer (CFD 1400 SS, Alpfrigo d.o.o., Logatec, Slovenia). The dry matter content of plant materials was in the range of 93.0–93.8%. The material was ground in a cutting mill (Retsch GM300) for 1 min at 1500 rpm and sieved using vibratory sieves (AS300 control, Retsch, Düsseldorf, Germany) with 1 mm Ø mesh elements for 4 min and the fraction obtained was ≤1 mm. Aqueous extracts (H_2_O) and 30% aqueous ethanolic extracts (30% EtOH) were prepared. Plant powders (0.50 g) were weighed into tubes and 5 mL of ultrapure water or 30% EtOH was added, mixed, and shaken at 60 rpm for 60 min on a Multi RS-60 Multirotator (Biosan, Riga, Latvia). Then, the samples were centrifuged, the supernatants were removed, and the procedure was repeated to obtain 1:20 (*w*/*v*) clear extracts of the plant materials in water and in 30% EtOH. The samples were prepared in duplicate and stored at −40 °C in a freezer until further experiments.

### 2.2. Chromatographic Analyses

The extracts were chromatographically analyzed using a 1290 Infinity system (Agilent Technologies, Waldbronn, Germany), coupled to an Agilent 6450 Q-TOF mass spectrometer equipped with a Jetstream ESI source and to an Agilent 1290 Infinity Diode Array Detector. 

The samples were subjected to a Zorbax 300SB-C18 column (2.1 × 150 mm; 5 µm; Agilent Technologies) kept at 40 °C. For the elution of the samples, a gradient of 5% water in acetonitrile (A) and 0.1% formic acid in water (B) was used as follows: 0–1.5 min 1.0% A, 21.50 min 25% A, 35.00–39.00 min 95% A, 39.10 min 1% A with the eluent flow rate set to 0.3 mL/min, regeneration time was 6 min with eluent flow rate set to 0.4 mL/min. The injection size was 2.0 µL. The mass spectrometer was working in negative ionization MS/MS mode with an MS acquisition rate of 1 spectrum/s and an MS/MS acquisition rate of 5 spectra/s. Fragmentor voltage was set to 135 V and the collision energy to 25 V. The diode array detector operated at a wavelength of 280 nm with 4 nm bandwidth. Data acquisition and initial data processing were carried out using the MassHunter software 10.0 (Agilent Technologies). Gallic acid was used to construct an external calibration graph for quantifying as gallic acid equivalent (GAE) using UV chromatograms at 280 nm. Compounds were identified by comparison of the m/z value, retention time, UV spectra, and MS/MS fragmentation patterns with standards or by comparing data from the literature or the METLIN database (Agilent Technologies). 

### 2.3. Determination of Antioxidative Properties

Analyses of antioxidative (AO) properties of plant extracts (1:20 *w*/*v*) in ultrapure water or in 30% EtOH were performed using the DPPH free-radical-scavenging method on an Infinite 200 Pro M Plex Mono Cuvette plate reader instrument (Tecan Austria Gmbh, Grödig, Austria).

Measurements were performed at 515 nm, using 50 µL of sample and 150 µL of DPPH (1 mM) solution per well. Ultrapure water and 30% EtOH were used as respective blank samples.

For quantification of AO values in Trolox and gallic acid (GA) equivalents, respective calibration curves were used, measured in the same conditions as samples. For finding the linear range of the samples, 1:200, 1:400, and 1:800 (*w*/*v*) sample dilution were measured in duplicate. For calculations, aqueous extracts’ 1:200 (*w*/*v*) dilution and 30% EtOH extracts’ 1:400 (*w*/*v*) dilutions were used. Results are expressed as AO values in Trolox equivalents and GA equivalents in mM, showing standard deviations of the duplicate measurements.

### 2.4. Determination of Antimicrobial Activity

Both Gram-positive (*Listeria monocytogenes* ATCC 13929, *Staphylococcus aureus* ATCC 25923) and Gram-negative (*Escherichia coli* NCCB 100282, *Campylobacter jejuni* ATCC 33291) bacterial strains were used in an in vitro study. The isolates originated from the bacterial strain collection of the Food Hygiene and Safety Division of the Estonian University of Life Sciences. The bacteria were cultured on nonselective Brucella blood agar (Oxoid, Basingstoke, UK) plates and incubated at 37 °C for 24 h, except for *C. jejuni* (at 42 °C for 24 h). One colony (1 µL) of each strain was transferred to 4 mL of a 0.9% NaCl solution, and turbidity was adjusted to 0.5 McFarland standard. The bacterial inoculum was adjusted to a concentration of 10^6^ colony-forming units per mL (cfu/mL).

The minimum inhibitory concentration (MIC) values of the extracts were determined against tested bacteria by the broth microdilution method in 96-well microplates, according to the EVS-EN ISO 20776-1:2020 [21] guidelines. Falcon^®^ sterile microplate with a clear flat bottom and TC-treated plates were used. For the MIC test, 100 µL of the plant extract was added to the first well of the microplate. Other wells were inoculated with 50 µL of Mueller–Hinton Broth (MHB, Oxoid, Basingstoke, UK). Two-fold dilution series of 1:1 to 1:512 were made to obtain concentrations ranging from 0.001 to 0.82 mg GAE/mL in aqueous extracts and 0.001 to 1.49 mg GAE/mL in ethanolic extracts depending on the contents of total polyphenols in the tested extracts. Column 1 of the microplate contained the highest concentration of extracts, while column 10 contained the lowest. Then, 50 µL of bacterial suspension was dispensed into each well of a sterile 96-well plate. The final concentration of the bacterial inoculum in each well was 5 × 10^5^ cfu/mL. Negative control wells (column 11) consisted of bacteria in MHB without extracts and positive control (column 12) contained gallic acid (1 mg GAE/mL). The plate was covered with a sterile lid and incubated for 18 h at 35 °C. The MIC value was defined as the lowest extract concentration able to completely inhibit visible growth of the target microorganism. Experiments were performed in triplicate and results were expressed as gallic acid equivalents per mg/mL (mg GAE/mL).

### 2.5. Statistical Analyses

Microsoft Excel 365 (Microsoft Corporation; Redmond, WA, USA) was used for data collection and statistical analyses including correlation analyses. The results were expressed as mean and standard deviation (SD). Correlations were considered strong if *r* ≥ 0.7, moderate if *r* > 0.3, and weak if *r* < 0.3 [22,23]. Statistical analysis was also performed using IBM SPSS Statistics software, Version 29.0 (IBM Corp., Chicago, IL, USA). A one-way ANOVA and Tukey HSD test were utilized to evaluate the significant differences (*p* < 0.05) between the tested berry and berry pomace extracts. 

## 3. Results and Discussion

### 3.1. Identification and Quantification of Phenolic Compounds

**Figure 1:**
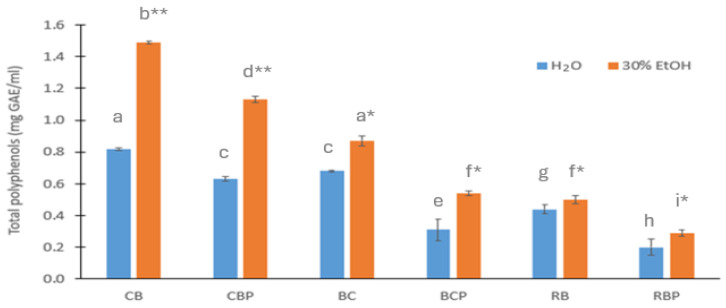
Total content of polyphenols (mg GAE/mL ± SD) in the aqueous (H_2_O) and ethanolic extracts (30% EtOH) of chokeberry berries (CB), chokeberry pomace (CBP), blackcurrant berries (BC), blackcurrant pomace (BCP), rowan berries (RB), and rowan berry pomace (RBP) determined by HPLC-DAD-UV. Columns with the same letters (a–i) do not differ significantly (*p* > 0.05). * *p* < 0.05, ** *p* < 0.01 compared to the H_2_O extract.

The total content of polyphenols in aqueous and ethanolic extracts of selected berries and berry pomaces is shown in Figure 1. The total content of polyphenols was higher in the ethanolic extracts than in the aqueous extracts and the difference between these extracts was statistically significant (*p* < 0.05). The content of total polyphenols was 1.49 ± 0.01 and 1.13 ± 0.02 mg GAE/mL in the ethanolic extracts of chokeberry berries (CB) and chokeberry pomace (CBP), respectively. Blackcurrant berries (BC) and pomace (BCP) and rowan berries (RB) and rowan berry pomace (RBP) contained 0.87 ± 0.01 and 0.54 ± 0.07 mg GAE/mL and 0.50 ± 0.03 and 0.29 ± 0.05 mg GAE/mL of polyphenols in the ethanolic extracts, respectively.

A total of 21 different polyphenolic compounds belonging to hydroxycinnamic acids, flavanols, flavonols, dihydrochalcones, and anthocyanins were tentatively identified from the extracts, of which 19 compounds were detected in BC and BCP extracts, and 16 compounds were found in both CB and RB and their pomace extracts (Table 1). Among hydroxycinnamic acids, protocatechuic acid was detected in all the studied extracts except for the ethanolic extract of RB. Caffeic acid was detected only in the extracts of BC and its pomace. Among flavanols, (epi)gallocatechins were found only in the extracts of BC and its pomace. A dihydrochalcone phloretin-di-C-hexoside was detected in all the tested extracts except for BC. Regarding anthocyanins, the most common compounds in the tested extracts were cyanidin hexosides. Delphinidin rutinoside and cyanidin rutinoside were detected only in the extracts of BC and BCP. Cyanidin pentosides were not found in the extracts of BC and its pomace or RB.

**Figure 2:**
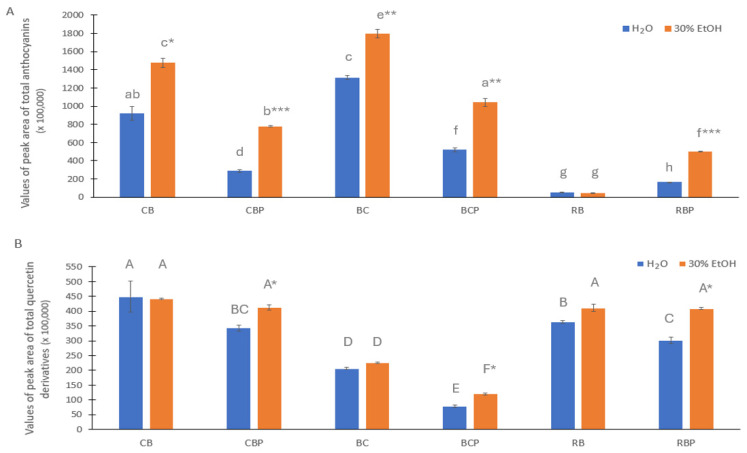
The extracted ion chromatogram peak areas (±SD) of total anthocyanins (**A**) and quercetin derivatives (**B**) obtained with water (H_2_O) and 30% aq. ethanol (EtOH) extraction for the berries and berry pomaces. Abbreviations: CB, chokeberry berries; CBP, chokeberry pomace; BC, blackcurrant berries; BCP, blackcurrant pomace; RB, rowan berries; RBP, rowan berry pomace. Columns with the same letters (a–h in (**A**) and A–F in (**B**)) do not differ significantly (*p* > 0.05). * *p* < 0.05, ** *p* < 0.01, *** *p* > 0.001 compared to the H_2_O extract.

Figure 2 shows the extracted ion chromatogram (EIC) peak areas of total anthocyanins and total quercetin derivatives in the aqueous and ethanolic extracts of the berries and their pomaces. The EIC peak areas of total anthocyanins were larger for ethanolic extracts compared to aqueous extracts (*p* < 0.05 except for RB). The EIC peak area of total anthocyanins was the largest in BC and CB samples followed by BCP and CBP. The EIC peak area of total quercetin derivatives was the largest in CB followed by CBP, RB, and RBP. Compared with tested berries, the differences in the EIC peak areas of total quercetin derivatives in aqueous and ethanol extracts were not significant (*p* > 0.05) for berry pomaces.

**Figure 3:**
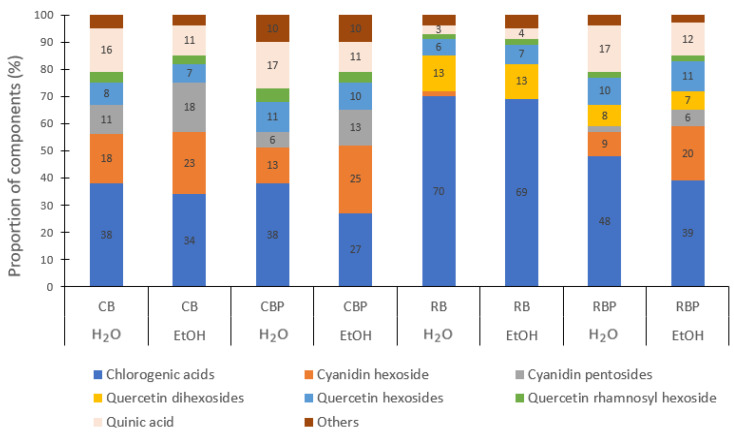
Polyphenolic profiles of chokeberry berries (CB), chokeberry pomace (CBP), rowan berries (RB), and rowan berry pomace (RBP) obtained with water (H_2_O) and 30% aq. ethanol (EtOH) extraction.

**Figure 4:**
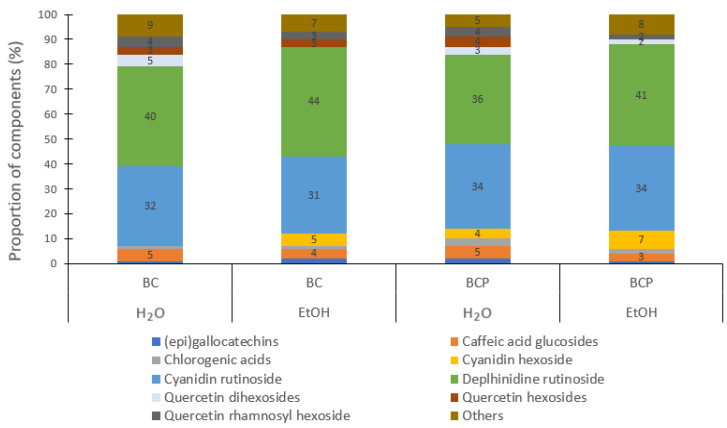
Polyphenolic profiles of blackcurrant berries (BC) and blackcurrant pomace (BCP) obtained with water (H_2_O) and 30% aq. ethanol (EtOH) extraction.

Polyphenolic profiles of selected berries and their pomaces obtained by extraction with water and 30% aq. ethanol are shown in Figure 3 and Figure 4. Chlorogenic acids, cyanidin hexoside 1, quercetin hexosides, quercetin rhamnosyl hexoside, and quinic acid were found in all the extracts of both CB and RB and their pomaces (Figure 3). 

In the aqueous and ethanolic extracts of CB, the proportion of identified polyphenolic compounds from highest to lowest were as follows: chlorogenic acids (34–38%) > cyanidin hexosides (18–23%) > cyanidin pentosides (11–18%) > quinic acid (11–16%) > quercetin hexosides (7–8%) > the rest of the compounds. A similar profile of polyphenols was also identified in CBP extracts, but the proportions of quercetin hexosides were higher (up to 3%) and the content of cyanidin pentosides was lower (up to 5%).

In both aqueous and ethanolic extracts of RB, the proportions of polyphenolic compounds from highest to lowest were similar: chlorogenic acids (69–70%) > quercetin dihexosides (13%) > quercetin hexosides (6–7%) > quinic acid (3–4%) > the rest of the compounds (Figure 3). A similar profile of polyphenols was also identified in RBP extracts, but the proportions of quinic acid (up to 14%), quercetin hexosides (up to 4%), and cyanidin hexoside 1 (up to 20%) were higher and content of chlorogenic acids was lower (up to 30%).

Polyphenolic profiles of BC and BCP obtained by extraction with water and 30% aq. ethanol are shown in Figure 4. A total of nine of the twenty-one detected compounds in the polyphenolic profiles of the aqueous and ethanolic extracts were higher in content than the other identified compounds according to the EIC peak areas (Figure 3). All nine compounds were identified in aqueous and ethanolic extracts except cyanidin hexoside 2 in berry aqueous extract, quercetin dihexoside in berry ethanolic extract, and quercetin hexosides in berry pomace ethanolic extracts. In the aqueous and ethanolic extracts of BC, the proportion of identified polyphenolic compounds from highest to lowest was as follows: delphinidin rutinoside (40–44%) > cyanidin rutinoside (31–32%) > quercetin rhamnosyl hexoside (3–4%) > quercetin hexosides (3%) > the rest of the compounds. A similar profile of polyphenols was also identified in BCP extracts, but the proportions of delphinidin rutinoside were lower (up to 4%) and that of cyanidin rutinoside was higher (up to 3%).

### 3.2. Antioxidative Properties

Antioxidative (AO) properties of the studied berries and their pomaces in 1:20 *w*/*v* extracts in water and 30% aq. ethanol are shown in Table 2. In the aqueous and 30% ethanolic extracts of berries and pomaces, the antioxidative properties from highest to lowest were as follows: CB > CBP > BC > BCP ≈ RB > RBP. The AO properties were statistically significantly (*p* < 0.05) higher in the ethanolic extracts than in the aqueous extracts. Rowan berry 30% ethanolic extract had equal AO properties to BCP 30% ethanolic extract (*p* ≥ 0.05). Regarding all tested plant materials, 30% ethanolic extracts (1:20 *w*/*v*) showed better antioxidative properties compared with extracts made with water. Correlation analysis revealed that AO had a strong positive correlation with anthocyanin content in aqueous extracts (*r* = 0.84) and moderately positive correlation in 30% ethanolic (*r* = 0.56) extracts. The content of quercetin derivatives had a weak positive correlation with AO (*r* = 0.1–0.14) both in aqueous and 30% ethanolic extracts. The total content of polyphenols, however, correlated with AO strongly, showing strong positive correlations both in aqueous (*r* = 0.92) and in 30% ethanolic extracts (*r* = 0.93).

### 3.3. Minimal Inhibitory Concentrations

In Table 3, the minimum inhibitory concentration (MIC) values of the tested berries and berry pomace extracts are presented for Gram-positive (G+) bacteria such as *L. monocytogenes* and *S. aureus*, as well as Gram-negative (G−) bacteria such as *E. coli* and *C. jejuni*. MIC results were expressed as gallic acid equivalents per mg/mL dry matter and gallic acid was positive control. The MIC value of pure gallic acid against the tested G+ bacteria was 0.02 mg GAE/mL (at a dilution of 1:64 of the extracts) and 0.03 mg GAE/mL (at a dilution of 1:32 of the extracts) for G− bacteria.

The sensitivity of tested microbes to the extracts of berries and berry pomaces starting from more sensitive bacteria was as follows: *S. aureus* > *L. monocytogenes* > *E. coli* ≈ *C. jejuni*. All tested berry extracts inhibited the growth of G+ bacteria such as *S. aureus* and *L. monocytogenes*. Among the berry pomaces, only BCP extracts had a similar effect against these G+ bacteria. G− bacteria such as *E. coli* and *C. jejuni* were inhibited by ethanolic extracts of berries and berry pomaces but not by aqueous extracts. Among all tested bacteria, *S. aureus* was the most sensitive to all tested aqueous and ethanolic extracts of berries and berry pomaces. CB showed the strongest antibacterial activity against *S. aureus* both in aqueous (at a dilution of 1:8) and ethanolic extracts (at a dilution of 1:16) with MIC values of 0.19–0.21 mg GAE/mL followed by BC with MIC values of 0.17–0.22 mg GAE/mL and CBP with MIC values of 0.28–0.32 mg GAE/mL. The remaining tested extracts showed an antibacterial effect only at the dilution of 1:2 or 1:4, where MIC values were 0.10–0.41 mg GAE/mL in aqueous extracts and 0.13–0.75 mg GAE/mL in ethanolic extracts depending on the total content of polyphenols (Figure 1) in the berries and their pomaces.

## 4. Discussion

The polyphenolic profile of berries and berry pomaces and their antibacterial effect have been investigated in several studies [10,18,19,20,24]. Chokeberry, blackcurrant, and rowan berries are rich sources of polyphenolic compounds [6,7,8]. In this study, the polyphenolic profile screening showed that tested berries and berry pomaces contained different polyphenols, e.g., hydroxycinnamic acids, flavanols, flavonols, dihydrochalcones, and anthocyanins. A total of 19 different polyphenolic compounds were detected in BC and its pomace extracts and 16 different compounds were found in both CB and RB and their pomace extracts. Therefore, BC and BCP were distinguished from other berries due to their more diverse composition of polyphenols. However, CB and RB and their pomace extracts had a similar polyphenolic profile and the proportion of chlorogenic acids was the highest in all these extracts. Polyphenolic profiles of CB and RB were similar in the study of Määttä-Riihinen et al. [25]. In this study, however, the EIC peak area of total anthocyanins was the largest in CB and BC samples, but the EIC peak area of total quercetin derivatives was the largest in CB and RB samples. The content and proportion of bioactive compounds in berries and berry pomaces are quite variable and they can vary depending on the berry variety, growing conditions, maturity and harvest time, and processing methods as well as climatic conditions [13,26].

In this study, both investigated extracts of chokeberry pomace contained approximately 77% of the total polyphenol content compared to the content in berries. Ethanolic extracts of blackcurrant and rowan berry pomaces contained 60% of the total polyphenol content of the respective berries. Therefore, according to our study, berry pomaces are a valuable source of polyphenols which has also been confirmed in several other studies [14,17]. Khanal et al. [27] found that after blueberry juice extraction, blueberry pomace contained 25% to 50% of the procyanidins compared to the content in fresh berries. In any case, the use of berry pomaces in food production deserves consideration for the development of new health-beneficial functional foods due to the valuable phenolic compounds [18]. Rowan berry extracts differed from other berries in this study, containing 1.5–3 times lower polyphenol levels than chokeberry or blackcurrant. This may be explained by the peculiarity of the rowan berry varieties and their low anthocyanin content. 

In this study, both water and 30% aq. ethanol (30% EtOH) extraction were used to obtain the best possible yield of soluble polyphenolic compounds in berries and berry pomaces. The optimal concentration of ethanol in solvent was established with preliminary tests. Other types of possible solvents were not used to have only a food-grade solvent system. Salaheen et al. [19] found that total phenolic content was higher in a 10% ethanolic extract compared to 10% methanol or water extracts. Bobinaitė et al. [17] found that the aqueous extract had the highest polyphenolic compound diversity compared to ethanolic or acetone extracts obtained from rowan berry pomace. In this study, the aqueous and ethanolic extracts generally had a similar diversity of polyphenolic compounds with some minor differences. Compared to other aqueous extracts, the aqueous extracts of BC and BCP had a more diverse composition of polyphenols, including caffeic acid, (epi)gallocatechins, delphinidin rutinoside, and cyanidin hexoside 2, which were not detected in other aqueous extracts. Protocatechuic acid and caffeic acid were present in BC and CB ethanolic extracts but were lacking in RB and RBP ethanolic extracts. The differences in the polyphenolic profiles can be explained by the fact that more hydrophobic compounds are extracted with a higher % of ethanol [7]. 

In this study, 30% ethanolic extracts showed better antioxidative properties for all tested berries and pomaces compared to water extracts, indicating that the addition of ethanol in the extraction solvent facilitates the extraction of compounds with better AO properties. The tested berries and pomaces, especially in the case of chokeberry and blackcurrant, had good antioxidant properties and this result is consistent with other similar studies [8,10,26]. Antioxidative properties had the strongest correlation with the total content of polyphenols (TPC) both in aqueous and 30% ethanolic 1:20 (*w*/*v*) extracts. Within the group of polyphenolic compounds, anthocyanins also had a strong correlation in aqueous (*r* = 0.84) extracts or a moderate positive correlation in 30% ethanolic (*r* = 0.56) extracts with AO. That result may be due to the fact that anthocyanins are semipolar compounds and thus also extractable with hydrophilic solvent, whereas in 30% ethanolic extracts the AO results were also due to more nonpolar compounds. Interestingly, quercetin derivates had a very weak correlation with AO properties, which can be explained by the fact that in plant matrices there is usually no or very little free quercetin, which is known as a very good antioxidant, whereas quercetin derivatives also present in the extracts in this study exhibit lower antioxidant properties as the free OH groups of the aglycone are substituted with glycosyl groups. This result is in accordance with the study of Lesjak et al. [28].

Berry additives in food can play a role in inhibiting the growth of microorganisms due to their potential antibacterial properties. In this study, MIC values were generally high, ranging from 0.10 to 0.75 mg GAE/mL, although concentrations as low as 0.001 to 0.09 mg GAE/mL were also found. The sensitivity of tested bacteria to the berry and berry pomace extracts was as follows: *S. aureus* > *L. monocytogenes* > *E. coli* ≈ *C. jejuni*. The antibacterial activity of the extracts against tested bacteria did not depend only on the TPC but probably also on other factors such as the concentration of organic acids in the extract. This was also confirmed in the study of Adamczak et al. [29]. The MIC tests showed that all the tested berry extracts of CB, BC, and RB inhibited the growth of *S. aureus* and *L. monocytogenes*, but only BCP had the same effect among pomaces against the above-mentioned G+ bacteria. Contrary to our results, Bobinaitė et al. [17] showed that the growth of G+ bacteria was also effectively suppressed by acetone, ethanolic, and aqueous extracts of rowan berry pomace. LaPlante et al. [24] found that cranberry extracts inhibited the growth of G+ bacteria, such as *Staphylococcus* spp., but not G– bacteria, such as *E. coli,* with MIC in the range of 0.02–5 mg/mL. In this study, CB and BC had strong antibacterial activity against *S. aureus,* which was inhibited by 8- and 16-fold dilution of aqueous and ethanolic extracts, respectively. All ethanolic extracts also inhibited the growth of G– bacteria, but the antibacterial effect only appeared when the extracts were diluted up to two or four times. Unlike the above, the growth of *E. coli* and *C. jejuni* was not inhibited by aqueous extracts of tested berries and berry pomaces. Similarly, Shirzadi Karamolah et al. [30] found that, in contrast to the alcohol extract, the aqueous extracts had no effect on the tested bacteria. In the present study, the antibacterial effect on the growth of the tested bacteria was also confirmed mainly by the ethanolic extracts. 

As previously known, the bioactive compounds found in berries are mainly phenolic compounds (phenolic acids, flavonoids, e.g., anthocyanins and flavonols, and tannins) [13] and they are associated with antibacterial properties of berries and berry pomaces [20]. Our previous studies [3,31] have shown good potential in using powdered plant material (including plant pomaces) as an antimicrobial agent in different products, ensuring the high microbiological quality of raw and cooked minced pork and marinated rainbow trout. Adamczak et al. [29] found that all tested individual pure compounds among flavonoids and organic acids showed antimicrobial properties, but their biological activity was moderate or relatively low against G+ and G− bacteria. Georgescu et al. [20] found that the extracted polyphenolic compounds from different berries showed an antibacterial effect on *E. coli, Bacillus subtilis*, and *S. aureus*. Furthermore, several studies have shown that procyanidins have an antibacterial effect against both G+ and G− bacteria [24,32]. The antibacterial mechanism of polyphenolic compounds is associated with the increased permeability of the pathogen’s outer membrane and plasma membrane, facilitating the entry of hydrophobic compounds into the bacterial cell by breaking the lipopolysaccharide layer [33]. However, G+ bacteria are more sensitive to flavonoids than G− bacteria due to differences in cell wall structure [34]. The target of flavonoids is the cell membrane of G+ bacteria by damaging its phospholipid bilayer structure and by inhibiting the bacterial respiratory chain and ATP synthesis [35].

In this study, the ethanolic extract of CB had the strongest antibacterial activity against *S. aureus* which could be explained with the highest content of total polyphenols found in this extract. However, the effect of BC and its pomace can be highlighted because both their aqueous and ethanolic extracts had antibacterial activity against *S. aureus* and *L. monocytogenes*, while no similar effect was detected for other pomaces against *L. monocytogenes*. This may be related to blackcurrant and its pomace having a more diverse polyphenolic composition and higher content of anthocyanins (e.g. delphinidin rutinoside, cyanidin hexoside 2, and cyanidin rutinoside) compared to other berries and their pomaces. In addition, the antibacterial effect may be related to the effect of organic acids and their derivatives on G+ and G− bacteria [36].

A study by Ma et al. [37] concluded that anthocyanins exhibit antibacterial properties by destroying the cell wall of foodborne pathogens. Furthermore, caffeic acid and (epi)gallocatechins were identified only in the extracts of BC and BCP in this study. Perumal et al. [33] found that caffeic acid and epicatechin 3-gallate from *Euphorbia hirta* have remarkable bactericidal effects against *Pseudomonas aeruginosa* by increasing the permeability of the bacterial outer membrane and plasma membrane. Catechins may also have antibacterial properties by reducing the antioxidant capacity of foodborne pathogens [37].

The studied berries are also a rich source of hydroxycinnamic acids. The most abundant hydroxycinnamate was chlorogenic acid, which is a complex of caffeic acid linked to quinic acid through an ester bond [26]. In this study, the highest proportion of chlorogenic acids was found in the extracts of CB and RB and their pomaces. Adamczak et al. [29] showed that chlorogenic acid had moderate activity against *E. coli,* but no activity against *Pseudomonas aeruginosa*. In this study, the antibacterial activity of chokeberry and rowan berries against *S. aureus* and *L. monocytogenes* was shown in both aqueous and ethanolic extracts, but not their pomaces, where the proportions of chlorogenic acids were lower.

In the polyphenolic profile, the highest proportion of quercetin derivatives was found in CB and RB ethanolic extracts and these extracts had an antibacterial effect on all the tested G+ and G− bacteria. The inhibition of pathogenic bacteria by quercetin is associated with damage to peptidoglycan structures [34]. No free quercetin was detected in the aqueous extracts of RBP, and these extracts showed no antibacterial activity against *L. monocytogenes, E. coli*, and *C. jejuni*. 

## 5. Conclusions

The study demonstrated a diverse profile of polyphenols in the extracts of chokeberry, blackcurrant, and rowan berry and their berry pomaces. Since the extracts of chokeberry and blackcurrant berries and berry pomaces contained a higher proportion of polyphenolic compounds, they also had better antibacterial and antioxidative properties compared to rowan berry and its pomace. In vitro antibacterial activity of tested extracts depended on the extraction solvent and mainly occurred for the ethanolic extracts. Findings suggest that chokeberry and blackcurrant berries and their pomaces are good sources of polyphenols with antioxidative properties and compounds that have antibacterial activity against selected bacteria. In practical applications, it is important to extract the valuable compounds from juice press residues for further usage in the composition of foods, especially for the enrichment of animal-origin foods. This kind of food valorization is in compliance with the zero-waste concept and goals of a circular bioeconomy.

## Figures and Tables

**Table 1:** Compounds tentatively identified by liquid chromatography–mass spectrometry from chokeberry, blackcurrant, and rowan berries and their pomace extracts.

Pseudomolecular ion Mass-to-Charge Ratio (*m*/*z*)	Compound	Detected in Aqueous Extracts	Detected in Ethanolic Extracts
191.0556	Quinic acid	+	+
**Hydroxycinnamic acids:**			
153.0193	Protocatechuic acid	+	CB, CBP, BC, BCP, RBP
337.0929	Coumaroylquinic acids	+	+
341.0878	Caffeic acid	BC, BCP	BC, BCP
353.0873	Chlorogenic acids	+	+
**Flavanols:**			
289.0718	Catechins	+	+
305.0700	(Epi)gallocatechins	BC, BCP	BC, BCP
577.1352	Procyanidin B type	+	+
**Flavonols:**			
301.0354	Quercetin	CB, CBP, BC, BCP, RB	+
433.0776	Quercetin pentosides	+	+
463.0882	Quercetin hexosides	+	+
595.1305	Quercetin pentosyl-hexosides	+	+
609.1461	Quercetin rhamnosyl hexoside	+	+
625.1410	Quercetin dihexosides	+	+
**Dihydrochalcones:**			
435.1297	Phloridzin	+	+
597.1825	Phloretin-di-C-hexoside	CB, CBP, BCP, RB, RBP	CB, CBP, BCP, RB, RBP
**Anthocyanins:**			
417.0827	Cyanidin pentosides	CB, CBP, RBP	CB, CBP, RBP
447.0928	Cyanidin hexoside 1	CB, CBP, RB, RBP	CB, CBP, RB, RBP
447.0928	Cyanidin hexoside 2	BC, BCP	BC, BCP
593.1506	Cyanidin rutinoside	BC, BCP	BC, BCP
609.1461	Delphinidin rutinoside	BC, BCP	BC, BCP

+, found in all tested extracts. Abbreviations: CB, chokeberry berries; CBP, chokeberry pomace; BC, blackcurrant berries, BCP, blackcurrant pomace; RB, rowan berries; RBP, rowan berry pomace.

**Table 2:** Antioxidative (AO) properties (±SD) of 1:20 (*w*/*v*) aqueous (A) and 30% ethanolic (B) extracts of berries and their pomaces.

Extracts	AO in Trolox eq mM (A)	AO in GAE mM (A)	AO in Trolox eq mM (B)	AO in GAE eq mM (B)
CB	9.08 ± 0.10 ^a^	0.004 ± 0.0000 ^A^	21.4 ± 0.16 ^b,^***	0.009 ± 0.0001 ^B,^**
CBP	5.55 ± 0.17 ^c^	0.003 ± 0.0001 ^C^	17.5 ± 0.48 ^d,^***	0.007 ± 0.0002 ^D,^**
BC	8.73 ± 0.19 ^a^	0.004 ± 0.0001 ^A^	16.0 ± 0.11 ^d,^**	0.007 ± 0.0000 ^E,^**
BCP	4.57 ± 0.34 ^c^	0.002 ± 0.0001 ^C^	13.0 ± 1.21 ^e,^*	0.005 ± 0.0005 ^F,^*
RB	3.61 ± 0.83 ^c^	0.002 ± 0.0004 ^C^	13.2 ± 1.57 ^e,^*	0.006 ± 0.0006 ^F,^*
RBP	0.57 ± 0.05 ^f^	0.001 ± 0.0000 ^G^	6.09 ± 2.56 ^c,^*	0.003 ± 0.0010 ^C,^*

Abbreviations: CB, chokeberry berries; CBP, chokeberry pomace; BC, blackcurrant berries, BCP, blackcurrant pomace; RB, rowan berries; RBP, rowan berry pomace. Columns with the same letters (^a–f^ in the Trolox and ^A–G^ in the GAE columns) do not differ significantly (*p* ≥ 0.05). * *p* < 0.05, ** *p* < 0.01, *** *p* > 0.001 compared to H_2_O extract.

**Table 3:** Minimum inhibitory concentration (MIC) (mg GAE/mL) of aqueous (A) and 30% ethanolic (B) extracts of berries and their pomaces against selected bacteria.

Extracts		*L. monocytogenes* G+	*S. aureus* G+	*E. coli* G−	*C. jejuni* G−	Min and Max MIC Values *
CB	A	0.41	0.21	-	-	0.001–0.82
	B	0.37	0.19	0.75	0.75	0.003–1.49
CBP	A	-	0.32	-	-	0.001–0.63
	B	0.28	0.28	0.57	0.57	0.002–1.13
BC	A	0.34	0.17	-	-	0.001–0.68
	B	0.22	0.22	0.22	0.22	0.002–0.87
BCP	A	0.16	0.16	-	-	0.001–0.31
	B	0.14	0.27	0.14	0.27	0.001–0.54
RB	A	0.22	0.11	-	-	0.001–0.44
	B	0.13	0.13	0.13	0.13	0.001–0.50
RBC	A	-	0.10	-	-	0.001–0.20
	B	0.15	0.15	0.15	0.15	0.001–0.29

* The highest and lowest possible MIC values of the tested extracts were determined at a dilution of 1:1 and 1:512, respectively. -, no antibacterial effect. Abbreviations: CB, chokeberry berries; CBP, chokeberry pomace; BC, blackcurrant berries, BCP, blackcurrant pomace; RB, rowan berries; RBP, rowan berry pomace.

## Data Availability

Data are contained within the article.
